# *Turrón* Coproducts as Source of Bioactive Compounds: Assessment of Chemical, Physico-Chemical, Techno-Functional and Antioxidant Properties

**DOI:** 10.3390/foods9060727

**Published:** 2020-06-03

**Authors:** José Manuel Lorente-Mento, Raquel Lucas-González, Estrella Sayas-Barbera, José Ángel Pérez-Álvarez, Juana Fernández-López, Manuel Viuda-Martos

**Affiliations:** IPOA Research Group, Agro-Food Technology Department, Escuela Politécnica Superior de Orihuela, Universidad Miguel Hernández de Elche, 03312 Orihuela, Alicante, Spain; jlorente@umh.es (J.M.L.-M.); raquel.lucasg@umh.es (R.L.-G.); estrella.sayas@umh.es (E.S.-B.); ja.perez@umh.es (J.Á.P.-Á.); j.fernandez@umh.es (J.F.-L.)

**Keywords:** almond, *turrón*, skins, coproducts, techno-functional, polyphenolic profile, antioxidant

## Abstract

The goals of this research were determined the proximate composition, physico-chemical, techno-functional properties, the polyphenolic profile, the organic acids and sugar content and the antioxidant capacities of flours obtained from almonds skins var. comuna (ASFC) and var. fritz (ASFF) coproducts produced in *Turrón* industry. The chemical composition and physico-chemical properties (pH, water activity and color) were determined. The water holding, oil holding and swelling capacities were also determined, as well as the polyphenolic profile. For the antioxidant capacity, four different assays were used namely: 2,2-diphenyl-1-picrylhydrazyl radical scavenging assay (DPPH^•^); Ferrous ions chelating activity (FIC); Ferric reducing antioxidant power (FRAP) and 2,2′-azinobis-(3-ethylbenzothiazoline-6-sulfonic acid) radical scavenging assay (ABTS^•+^). The flours obtained from ASFC and ASFF had a high content of dietary fiber (70.50 and 69.83 g/100 g, respectively). The polyphenolic profile, determined by High Performance Liquid Chromatography, identified 21 and 19 polyphenolic compounds in both ASFC and ASFF, being epicatechin and catechin the most abundant compounds. In reference to the antioxidant capacity regards, with all methods assayed except FRAP, ASFC had higher antioxidant activity than ASFF. These coproducts show good technological and antioxidant properties, which makes them a good alternative for its use in the development of new foods.

## 1. Introduction

The seeds produced be almond tree (*Prunus dulcis* (Mill.) D.A. Webb) family *Rosaceae*, are usually consumed crude, toasted or fried although they could also be part, like ingredient, of diverse foods for example ice creams, desserts sauces as well as typical Spanish sweets such as *turrón* and *mazapán*. 

Actually, in Spain, almost 32,000 t *turrón* and *mazapán* are destined to the national market every year, while for exports are destined approximately 5000 t. As mentioned above, *turrón* and *mazapán* are typical Spanish sweet products elaborate using as ingredients almonds, sugar and honey and manufactured in traditional ways. The almond content varies depending on the type of *turrón* but it is ranged between 45 and 65%. For *mazapán* the content of almond must be 50%. To elaborate these confectionery products, skinless almond must be used. Thus, skinless almonds usually are achieved by separating the tegument by soaking and then, they are peeling without any thermal treatment [1]. This fraction represents between 4 and 8% of almond weight.

In view of these data, the *turrón* and *mazapán* industry generate a great volume of coproducts which are in general utilized for cattle feed or as crude material to obtain energy. As mentioned Martín-Sánchez et al. [2] the discard of these products generally signifies a problem and could have undesirable effects on the environment. Consequently, new features in relation to the utilization of these coproducts for further exploitation on the creation of food ingredients with great nutritional value have showed increasing interest. Nevertheless, over the past few years, numerous research projects have been undertaken to evaluate the possible use of these coproducts as sources of bioactive compounds [3]. In this way, polyphenols such as flavonols, flavanols and hydroxycinnamic acids, found in almond skins could have a role in reducing risk factors against chronic inflammatory disorders and ageing illnesses [4]. Furthermore, as mentioned Monagas et al. [3] almond skins are rich source of antioxidant dietary fiber, which is recognized as a useful ingredient for the control of oxidative processes in food products as well as a potential functional food ingredient. Additionally, antioxidant dietary fiber showed (i) important therapeutic implications for certain health conditions such as diabetes, cardiovascular diseases and certain type of cancer, aging and degenerative processes [5] and (ii) from a techno-functional point of view fiber has shown properties, such as water and oil holding capacity, swelling capacity, emulsifying activity and gel formation [6]. Then, these coproducts have a potential use to isolate specific bioactive compounds for their application in various purposes like nutraceutical supplements, dietary additives, new ingredients and novel pharmaceutical products, contributing to the recovery of agro-industrial process waste, with major industrial, economic and environmental impact as informed Ayala-Zavala et al. [7]. Nevertheless, it must be taken into account that the recovery of bioactive substances from natural resources, by either conventional or emerging technologies, must be clearly characterized by several different stages when the recovery goals are high added-value compounds: macroscopic pre-treatment; separation of macromolecules and micromolecules; extraction; separation and purification; and finally product development [8,9].

Therefore, it is essential to analyses these coproducts to identify changes in the process and reusing the new product obtained, overcome environmental problems, and finally add a value to these products. In this way, the aim of this research was determined: the proximate composition as well as the physico-chemical and techno-functional properties; the polyphenolic profile; the organic acids and sugar content and the antioxidant capacities of flours obtained from almonds skins coproducts produced in *Turrón* industry to establish its use as potential ingredient in the development of novel food products.

## 2. Materials and Methods

### 2.1. Plant Material

Almond skins from two varieties: cv. comuna and fritz were obtained as coproduct from a *Turrón* industry situated in Jijona (Spain). This industry used peeled almond and honey as main ingredients to elaborate this product.

### 2.2. Sample Preparation

Almond skins were dried at 45 °C during 5 h in an air tunnel drier. Then, a grinder mill and sieves were used to obtain flours with a particle size of less than 0.417 mm.

### 2.3. Chemical Composition

The proximate composition (fat, ash, protein and moisture content expressed as g/100 g dry weight) of almond skin flours was analysed by means of the pertinent Association of Official Agricultural Chemists (AOAC) methodologies [10]. Total dietary fiber and insoluble dietary fiber were analyzed according to AOAC assays [10]. Soluble dietary fiber was determined by subtracting the insoluble dietary fiber fraction from the Total dietary fiber.

### 2.4. Physico-Chemical Properties

The pH of samples was determined in a suspension obtained from blending 10 g of almond skin flour with 100 mL of deionized water for 5 min, using a pH-meter pH/Ion 510. The water activity (Aw) was determined by means of a hygrometer NOVASINA TH-200 (Novasina; Pfaffikon, Switzerland) at 25 °C. The color parameters were analyzed in the CIEL*a*b* color space using a Minolta CM-700 (Minolta Camera Co., Osaka, Japan)with the next settings: illuminant D_65_, observer angle 10°, SCI mode, 11 mm aperture for illumination and 8 mm for measurement. The CIEL*a*b* coordinates analyzed were lightness (L*), redness (a*) and yellowness (b*), from which the psychophysical magnitudes hue and Chroma were obtained.

### 2.5. Techno-Fuctional Properties

The Water holding capacity (g water/g almond skin), oil holding capacity (g oil/g almond skin) and swelling capacity (mL/g almond skin) were analyzed following the methodology reported by Vázquez-Ovando et al. [11]. Emulsion capacity and emulsion stability were evaluated following the recommendations of Chau et al. [12]. The emulsion capacity was expresses as the volume of emulsified layer/volume of whole layer in the centrifuge tube ×100 while the emulsion stability was expressed as the volume of remaining emulsified layer/original emulsion volume ×100.

### 2.6. Organic Acid and Sugar Content

Two grams of each almond skin flours were mixed with 20 mL of ortho-phosphoric acid in water (0.1:99.9, *v/v*) using an IKA T25 homogenizer ((IKA, Staufen, Germany)) for 40 s at 13,500 r.p.m. and centrifuged at 7500× *g*, during 25 min at 4 °C. The supernatants were filtered through 0.45 μm membrane filters and determined by High Performance Liquid Chromatography in HP-1100 instrument coupled with two detectors: DAD G-1315A (set at 210 nm) and RID G1362A. Samples (20 μL) were injected into a Supelcogel C-610 H column (30 cm × 0.78 cm) at 30 °C using as mobile phase ortho-phosphoric acid in water (0.1:99.9, *v/v*) with a gradient elution at 0.5 mL/min, during 35 min [13]. The organic acids and sugars were identified and quantified by comparison with the standards previously injected in the same conditions.

### 2.7. Total Phenolic Content, Total Flavonoid Content and Tannins Content

The total phenolic contents of the almonds skins were quantified using Folin-Ciocalteu’s reagent [14]. Results were expressed in terms mg gallic acid equivalents per grams of almond skin. The total flavonoid content was measured using the colorimetric method with aluminum chloride [15]. Results were expressed as mg rutin equivalents/g sample. The tannins content was determined employing the methodology reported by Price et al. [16]. The results were expressed in terms of mg catechin equivalents/g of almond skin.

### 2.8. LC-ESI-MS/MS Characterization

Almond skin flours characterization was performed using a Nexera XR-UHPLC equip (coupled with a Qtrap 4500 fitted with a heated electrospray ionization source (ESI V-source) following the specification of Pellegrini et al. [17]. The separation module was equipped with a ACE Excel 2 C_18_-PFP column (100 mm × 0.21 cm, ID 2 μm). The mobile phases were formic acid in water (0.1:99.9, *v*/*v*) as solvent A and acetonitrile as solvent B with a gradient elution at 0.3 mL/min. Peak areas for the selected ions were determined by mean of Multi-Quant software from Sciex while the quantitation was done by the additional standard method. Table S1 of supplementary data showed the precursor ions (*m*/*z*), fragment ion transitions and main UHPLC-MS/MS optimized parameters (DP: declustering potential; EP: entrance potential; CE: collision energy; CXP: collision cell exit potential). Values were expressed as µg/g almond skin.

### 2.9. Antioxidant Activity

#### 2.9.1. 2,2-Diphenyl-1-Picrylhydrazyl Radical Scavenging Assay (DPPH)

DPPH^•^ free radical scavenging capacity was determined by the methodology proposed by Brand-Williams et al. [18]. Results were expressed as mg Trolox Equivalents (TE)/g of almond skin.

#### 2.9.2. Ferric Reducing Antioxidant Power (FRAP)

Ferric reducing antioxidant power was determined following the recommendations of Oyaizu [19]. Results were expressed as mg TE/g of almond skin.

#### 2.9.3. 2,2′-Azinobis-(3-Ethylbenzothiazoline-6-Sulfonic Acid) Radical Scavenging Assay

Radical cation (ABTS^•+^) scavenging activity assay was determined using the method proposed by Gullón et al. [20]. Results were expressed as mg TE/g of almond skin.

#### 2.9.4. Ferrous Ion-Chelating Capacity Assay (FIC)

Ferrous ion-chelating capacity was measured by inhibiting the formation Fe^2+^-ferrozine complex according to the method reported by Mahdavi et al. [21]. Results were expressed as mg EDTA/g of almond skin.

### 2.10. Statistical Analysis

All experiments were carried out in triplicate and data are reported as mean ± standard deviation. The statistical analyses were made using Statgraphics 5.1 for Windows. The differences of mean values among proximate composition, physico-chemical, techno-functional, organic acids and sugars content, total phenolic, total flavonoid and tannins contents as well as the antioxidant properties were analyzed by one-way analysis of variance (ANOVA). To ascertain whether there were significant differences (*p* < 0.05) between samples, contrasts between means were made using the Tukey’s test.

## 3. Results

### 3.1. Chemical Composition

Table 1 showed the chemical composition of almond skins flours obtained from varieties comuna (ASFC) and fritz (ASFF). For moisture and ash content no significant statistically differences (*p* > 0.05) were obtained between ASFC and ASFF. Nevertheless, ASFF had higher protein and lower lipid content (*p* < 0.05) than ASFC.

The results obtained for protein content are so close to those reported by Mandalari et al. [22] in almond skins cultivated in California (103 mg/g). However, for fat content the values are lower than those reported by the same authors (22.2 g/100 g) or those reported by Pasqualone et al. [23] who found a fat content in almond skins of 21.9 g/100 g. Table 1 also showed the total dietary fibre insoluble dietary fibre and soluble dietary fibre contents of ASFC and ASFF, as well as, the ratio between insoluble dietary fibre and soluble dietary fibre. ASFF and ASFC had a total dietary fibre content of 69.83 and 70.50 g/100 g dry matter respectively, without statistical significant differences (*p* > 0.05) between samples. The values obtained were higher than those reported by Mandalari et al. [24] who reported a total dietary fibre content obtained from almond skin flour extracted with a new freeze-thawing methodology of 45.10 g/100 g dry matter. The total dietary fibre obtained are compose basically by insoluble dietary fibre (66.06 and 69.86 g/100 g dry matter for ASFF and ASFC, respectively). It should be noted that flours obtained from almond skins had an insoluble-soluble ratio of 17.56 and 44.78 (*p* < 0.05) for ASFF and ASFC, respectively.

It is very important, for a product which could be considered rich in fibre, to show an ideal balanced between insoluble and soluble dietary fibre due to soluble fiber provide high hydration capability and swells to form viscous solutions, whilst isoluble fibre has the property to adsorb and maintain water into their matrix [25]. Flours obtained from agro-foods coproducts, which have in their composition high content in dietary fibre, may be utilized as potential functional ingredients thanks to these flours offer several health benefits including their capacity to reduce LDL-cholesterol and total cholesterol levels, increase glucose tolerance as well as the insulin response, reduction of hypertension and hyperlipidaemia, contribute to gastrointestinal health and the prevention of several cancers [6].

### 3.2. Physico-Chemical and Techno-Fuctional Properties

The physico-chemical properties of ASFC and ASFF are given in Table 2. One of the most important parameters to be analyzed when studying the physico-chemical properties of flours obtained from co-products is pH. The pH plays a significant role in the possible incorporation of these flours, as ingredients, in different food matrices. Low pH values may destabilize the matrices which they are added by precipitation or denaturation of the proteins. Additionally, pH can influence several techno-functional properties of flours like: water or oil holding capacity, foam capacity, and so on. Both ASFF and ASCF showed a slightly acidic pH, being higher (*p* < 0.05) in ASFF than in ASCF.

The water activity values (Table 2) of ASFC and ASFF were 0.123 and 0.121 respectively, without statistical differences (*p* > 0.05) between samples. The low water activity is widely associated with product degradation. This low value point to that the risk of degradation, due to microorganism, enzymes or non-enzymatic reactions, is marginal [26]. Another important parameter from the physical-chemical point of view is color. Therefore, the addition of flours, as potential food ingredients, cannot significantly affect the color of the product into which it is incorporated or/and influence the way in which it is accepted by the potential consumer. For all color parameters (lightness, redness, yellowness, hue and Chroma) ASFC showed higher values (*p* < 0.05) than ASFF (Table 2). For lightness, the results obtained were in agreement with Guiné et al. [27] who reported L* values for almonds skins cultivated in United States, Spain and Portugal ranged between 40 and 49. However, these authors reported values for redness (a*) and yellowness (b*) higher than those obtained in this work. Similarly, Pasqualone et al. [23] also reported values for the coordinates redness and yellowness higher than those obtained in this work. This fact could be explained by the used, in these studies, of higher temperatures than used in this work to dry the skins as well as the occurrence of Maillard reaction during thermal treatment and the enzymatic oxidation of phenolics to brown quinones due to air exposure [28].

The techno-functional properties of almond skin flours were showed in Table 2. ASFF had higher water holding capacity (*p* < 0.05) than ASFC. In contrast, ASFC had higher values (*p* < 0.05) for oil holding capacity and emulsion capacity than ASFF. For swelling capacity and emulsion stability no statistical differences were obtained (*p* > 0.05) among ASFC and ASFF. The Techno-functional properties of flours obtained from several coproducts depend of several factors like the chemical structure of constituents, porosity, particle dimension, ionic form, extraction conditions, pH values, insoluble: soluble dietary fiber ratio, etc. [29].

### 3.3. Organic Acid and Sugars Content

**Figure 1:**
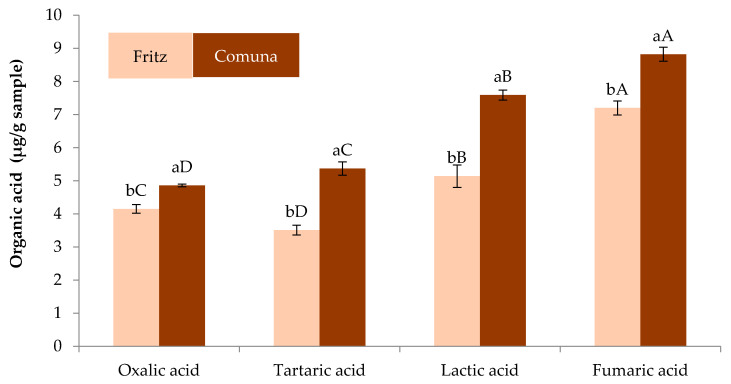
Organic acids content of almond skin flours obtained from Turrón coproducts. For the same compound, bars followed by different lowercase letter are significantly different (*p* < 0.05) according to Tukey’s Multiple Range Test. For the same almond skin bars followed by different capital letter are significantly different (*p* < 0.05) according to Tukey’s Multiple Range Test.

**Figure 2:**
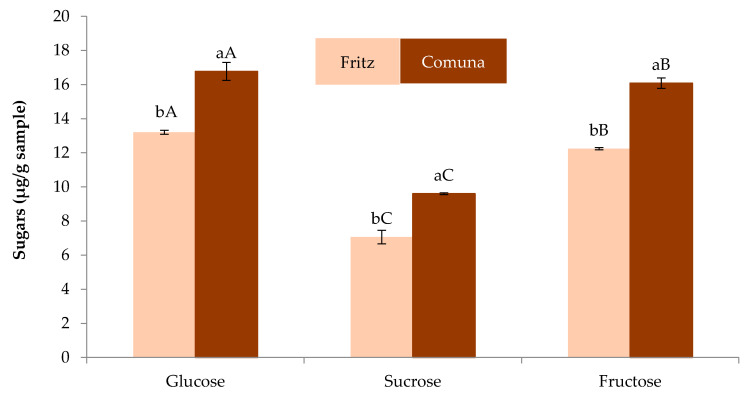
Sugar content of almond skin flours obtained from Turrón coproducts. For the same compound, bars followed by different lowercase letter are significantly different (*p* < 0.05) according to Tukey’s Multiple Range Test. For the same almond skin bars followed by different capital letter are significantly different (*p* < 0.05) according to Tukey’s Multiple Range Test.

The organic acid and sugar content of almond skins flours obtained from varieties comuna (ASFC) and fritz (ASFF) is given in Figure 1 and Figure 2, respectively. In both ASFC and ASFF four organic acids were found (Figure 1). For all organic acids identified ASFC had higher contents (*p* < 0.05) than ASFF. In ASFC fumaric acid was present in higher (*p* < 0.05) concentration than other organic acids following by lactic, tartaric and oxalic whereas in ASFF tartaric acid was found in lowest concentration being fumaric acid the main (*p* < 0.05) organic acid detected.

As regards sugars content (Figure 2), sucrose, glucose and fructose were found as sugar constituents in almond skins flours obtained from varieties comuna and fritz. The sugar concentration in ASFC was higher (*p* < 0.05) than ASFF. The main sugars found in ASFC were glucose and fructose without statistical differences (*p* > 0.05) between them while sucrose was found in lowest concentration. In ASFF, glucose was found in highest concentration (*p* < 0.05) followed by fructose and sucrose with statistical difference (*p* < 0.05) between them. The values found in this work were in opposite with Saura-Calixto et al. [30] who informed that almond skins contained principally sucrose and raffinose, a low concentration of glucose, fructose and inositol, and traces of arabinose and xylose. However, Mandalari et al. [31] reported that the main sugars found in blanched skins were glucose, arabinose and xylose, with values higher than those obtained in this work.

### 3.4. Total Phenolic, Total Flavonoid and Tannins Content

The total phenol content (TPC), total flavonoid content (TFC) and tannin content (TC) of extracts from almond skins flours obtained from varieties comuna (ASFC) and fritz (ASFF) are presented in Table 3.

ASFF had lower TPC and TFC than ASFC with statistical significant differences (*p* < 0.05) between them. The TPC values of both ASFC and ASFF were higher than those obtained Bolling et al. [32] in almond skins obtained from nonpareil, carmel, butte, sonora, fritz, mission and monterey cultivars harvested in California with values comprise between 58 and 159 mg gallic acid equivalents/100 g sample. However, Garrido et al. [33] reported that the total phenols content present in the almonds skins obtained from cultivars of Spain and America varied from 0.91 to 3.21 g/100g. These values were higher than those reported in this work. These differences could be explaining to cultivar, enviromental conditions and geography differences. Valdés et al. [34] analyzed the total flavonoid content of skins obtanied from some almond varieties: three from Spain and four from America. These authors found a total flavonoids content varied from 0.46 to 1.16 mg/g of almond skin which is lower than those obtained in this work. For tannin content, again ASFC showed higher values (*p* < 0.05) than ASFF. The results obtained in this study were similar than those found by Garrido et al. [33] in almond skins obtained from cultivars of Spain and America with values comprised between 5.81 and 43.3 mg/g of peel powder.

### 3.5. Polyphenolic Profile

Numerous studies informed that almond skins contains around 60.0–80.0% of polyphenolic compounds presents in almond seed. It should be noted that these polyphenolic compounds existing in the almond skin could be largely responsible for the high antioxidant capacity showed for such materials [3,24,35]. The polyphenolic profile of ASFC and ASFF is given in Table 4. The mean value of polyphenolic compounds identified was 273.44 μg/g for ASFC and 95.93 μg/g for ASFF with statistically significant differences (*p* < 0.05) between samples.

For ASFC, twenty-one polyphenolic compounds were detected and quantified. The polyphenolic profile was composed by 14.28% hydroxybenzoic acids (three compounds), 38.10% hydroxycinnamic acids (eight compounds) 9.52% flavanols (two compounds) and 38.10% flavonols (eight compounds). In ASFF, nineteen compounds were found. The polyphenolic profile was composed by 15.79% hydroxybenzoic acids (three compounds), 36.84% hydroxycinnamic acids (seven compounds), 10.53% flavanols (two compounds) and 36.84% flavonols (seven compounds). The main polyphenolic compounds found in ASFC samples were (*p* < 0.05) epicatechin, catechin, kaempherol-3-glucoside and chlorogenic acid with values of 101.89, 96.54, 27.00 and 8.30 µg/g of flour respectively. The principal compounds identified in the ASFF extract were epicatechin, catechin, 4-hydroxybenzoic acid and kaempherol-3-glucoside with values of 31.97, 26.61, 12.02 and 8.30 µg/g of flour (dry weight, d.w.) respectively. Regarding the principal polyphenolic compounds found in almond skins there are contrary values in the scientific literature. Monagas et al. [3] informed that the most representative polyphenolic compounds found in almond skins obtained from Spanish varieties were catechin (90.1 µg/g d.w.), epicatechin (36.6 µg/g d.w.) and isorhamnetin-3-rutinoside (27.6 µg/g d.w.) while in American varieties the main compounds were isorhamnetin-3-rutinoside, catechin and kaempferol-3-rutinoside with values 41.4, 36.4 and 31.8 µg/g d.w., respectively. Mandalari et al. [24] reported that the main compounds found in almonds skins var. maisie Jane’s cultivated in California were isorhamnetin-3-rutinoside (53.79 µg/g d.w.), catechin (50.57 µg/g d.w.) and kaempferol-3-rutinoside (40.76 µg/g d.w.). In more recent study Smeriglio et al. [36] analyze the polyphenolic profile of almond skin var. pizzuta. These authors mentioned that naringin (216.28 µg/g fresh weight, f.w.), kaempferol-3-rutinoside (108.29 µg/g f.w.) and kaempferol-3-glucoside (40.8 µg/g f.w.) were the principal polyphenolic compounds found in the skins. Prgomet et al. [37] analyzed the polyphenolic profile of almond skins cultivated in Protugal. These authors found that the main compounds were naringenin-7-O-glucoside, isorhamnetin-3-O-rutinoside and catechin with values of 9.90, 7.01 and 4.28 µg/g d.w., respectively. To explain this great variability in the results obtained in almond skins polyphenolic profile, its very important to notice that the polyphenolic type and concentration is influenced by numerous aspects including, variety, environmental aspects, location and altitude, agronomic works, ripeness stage as well as, extraction, identification methods, etc. [38].

### 3.6. Antioxidant Activity

The antioxidants found in several coproducts, as informed Wijeratne et al. [39], could provide protection against oxidative deterioration following several mechanisms such as reducing oxygen content, interrupting singlet oxygen, avoiding first-chain initiation by scavenging initial radicals, binding of metal ion catalysts and chain breaking to avoid continuous hydrogen elimination from substrates. To determine the antioxidant properties of almond skins flours obtained from varieties comuna (ASFC) and fritz (ASFF), four different assays were used. Table 5 showed the results achieved for the antioxidant properties of ASFC and ASFF.

In the DPPH^•^ and ABTS^•+^ assays ASFC had a higher (*p* < 0.05) capacity to inhibit DPPH and ABTS radicals than ASFF. As regards the FRAP assay no statistical differences (*p* > 0.05) were found among ASFC and ASFF samples (Table 5). For FIC assays, once more the extract from ASFC sample had higher (*p* < 0.05) chelating activity than ASFF extract.

In the scientific literature there are several works that reported the antioxidant capacity of almond skins achieved as coproducts have been analyzed. Valdes et al. [34] analyzed the antioxidant activity of seven almond cultivars: 3 cultivars from Spain namely marcona, guara and planeta and 4 cultivars from America such as butte, colony, carmel and padre using the DPPH and FRAP assays. For FRAP assay, they found values varied between 382 and 556 µMol TE/g which were lower than those obtained for ASFF and ASFC. Regarding to DPPH assay, a great radical scavenging activity (values comprised between 90 and 93%) was obtained for all samples analyzed. Similarly, Smeriglio et al. [36] analyze the antioxidant activity of natural and blanched skins obtained from almond var. pizzuta by means of ABTS^•+^, FRAP and FIC assays. For ABTS^•+^ assay, values very similar than ASFC were obtained for natural skins, while blanched skins had values similar than ASFF. However, for FIC and FRAP assay the natural and blanched skins obtained from almond var. pizzuta had higher values than obtained in this work.

Polyphenolic compounds existing in almond skins could be considered responsible for antioxidant activity, essentially due to these compounds showed their antioxidant properties by numerous possible action mechanisms, including free radical scavenging capacity, hydrogen donors, chelation activity of transition metals and singlet oxygen quenching capacity [40]. The additive and synergistic effects of such bioactive compounds, found in almond skins flours may be responsible for their strong antioxidant capacities. In this sense, the scientific literature reported numerous studies where a high correlation among polyphenolic compounds content and antioxidant activity was achieved [17,41].

## 4. Conclusions

With the results found in this study, the utilization, as potential food ingredient, of almond skins flours obtained from coproducts produced in *Turrón* industry should be taken into account due to several reasons such as: these coproducts show an elevate content in insoluble and soluble dietary fiber and polyphenolic compounds (principally hydroxycinnamic acids and flavanols) which could have numerous beneficial effects in human health. Additionally, these coproducts show good technological and antioxidant properties, which makes them a good alternative for its use in the development of functional foods.

## Figures and Tables

**Table 1:** Proximate composition of almond skin flours obtained from *Turrón* coproducts.

Sample	Moisture	Protein	Fat	Ash	Total Dietary Fibre	Insoluble Dietary Fibre	Soluble Dietary Fibre	IDF/SDF
ASFF	3.37 ± 0.61 ^a^	13.29 ± 0.41 ^a^	13.96 ± 0.31 ^b^	4.71 ± 0.19 ^a^	69.83 ± 3.39 ^a^	66.06 ± 0.63 ^b^	3.76 ± 0.85 ^a^	17.56 ± 0.42 ^b^
ASFC	3.82 ± 0.68 ^a^	11.77 ± 0.09 ^b^	16.90 ± 0.04 ^a^	4.23 ± 0.81 ^a^	70.50 ± 2.05 ^a^	69.86 ± 0.16 ^a^	1.56 ± 0.89 ^b^	44.78 ± 0.31 ^a^

Values expressed as: g/100 g almond skin flour. IDF/SDF: insoluble and soluble dietary fibre ratio. Values with different lowercase letter in the same column indicates significant differences (*p* < 0.05). ASFC: almond skins flours obtained from variety comuna; ASFF: almond skins flours obtained from variety fritz.

**Table 2:** Physico-Chemical and Techno-functional properties of almond skin flours obtained from Turrón coproducts.

Physico-Chemical Properties										
Sample	pH	Aw		Color Coordinates						
				Lightness	Redness	Yellowness		Chroma		Hue
ASFF	6.06 ± 0.09 ^a^	0.123 ± 0.002 ^a^		43.90 ± 0.48 ^b^	6.24 ± 0.24 ^b^	7.40 ± 0.32 ^b^		9.68 ± 0.39 ^b^		49.86 ± 0.21 ^b^
ASFC	5.77 ± 0.01 ^b^	0.121 ± 0.001 ^a^		49.34 ± 1.26 ^a^	8.48 ± 0.39 ^a^	13.74 ± 0.85 ^a^		16.15 ± 0.92 ^a^		58.31 ± 0.51 ^a^
**Techno-Functional Properties**										
**Sample**	**Water Holding Capacity** **(g Water/g Flour)**		**Oil Holding Capacity** **(g Oil/g Flour)**		**Swelling Capacity** **(mL/g Flour)**		**Emulsifying Capacity** **(mL/100 mL)**		**Emulsion Stability** **(mL/100 mL)**	
ASFF	4.97 ± 0.11 ^a^		1.81 ± 0.09 ^b^		2.97 ± 0.75 ^a^		67.77 ± 2.04 ^b^		100.0 ± 0.00 ^a^	
ASFC	4.11 ± 0.09 ^b^		2.08 ± 0.08 ^a^		2.34 ± 0.42 ^a^		76.66 ± 4.71 ^a^		100.0 ± 0.00 ^a^	

For the same parameter or property, different lowercase letter indicates significant differences (*p* < 0.05). Aw: The water activity.

**Table 3:** Total phenolic, total flavonoid and tannins content of almond skin flours obtained from *Turrón* coproducts.

Sample	TPC (mg Gallic Acid Equivalents/g Flour)	TFC (mg Rutin Equivalents/g Flour)	TC (mg Catechin Equivalents/g Flour)
ASFF	1.64 ± 0.14 ^b^	8.23 ± 0.37 ^b^	8.53 ± 0.17 ^a^
ASFC	6.39 ± 0.41 ^a^	17.08 ± 0.94 ^a^	30.13 ± 0.50 ^a^

For the same assay different lowercase letter indicate significant differences (*p* < 0.05). TPC: total phenol content, TFC: total flavonoid content, TC: tannin content.

**Table 4:** Polyphenolic profile of almond skin flours obtained from *Turrón* coproducts.

Compound	ASFC	ASFF
Protocatechuic acid	6.46 ± 0.06 ^eB^	6.64 ± 0.04 ^eA^
4-Hydroxybenzoic acid	6.43 ± 0.09 ^eB^	12.02 ± 0.18 ^cA^
Catechin	96.54 ± 0.77 ^bA^	26.61 ± 1.12 ^bB^
Vanillic acid	4.13 ± 0.05 ^fA^	3.18 ± 0.16 ^fB^
Caffeic acid	0.56 ± 0.01 ^jA^	0.24 ± 0.01 ^jB^
Ferulic acid	1.72 ± 0.02 ^iA^	0.68 ± 0.03 ^hB^
*O*-coumaric	0.04 ± 0.01 ^mA^	0.04 ± 0.00 ^lA^
Sinapic acid	0.37 ± 0.01 ^kA^	0.38 ± 0.02 ^iA^
Syringic acid	0.03 ± 0.00 ^mB^	0.48 ± 0.07 ^iA^
Cinnamic acid	0.24 ± 0.02 ^l^	ND
Chlorogenic acid	8.30 ± 0.92 ^dA^	0.40 ± 0.02 ^iB^
Epicatechin	101.89 ± 4.48 ^aA^	31.97 ± 0.51 ^aB^
*p*-coumaric	0.35 ± 0.01 ^kA^	0.14 ± 0.02 ^kB^
Quercetin-3-rutinoside	9.52 ± 0.19 ^dA^	1.59 ± 0.22 ^hB^
Quercetin-3-glucoside	0.38 ± 0.00 ^m^	ND
Kaempferol-3-rutinoside	27.00 ± 0.77 ^cA^	7.19 ± 0.52 ^dB^
Kaempferol-3-glucoside	0.11 ± 0.01 ^mB^	0.19 ± 0.01 ^kA^
Isorhamnetin-3-rutinoside	0.08 ± 0.01 ^mA^	0.08 ± 0.01 ^lA^
Quercetin	2.49 ± 0.01 ^hA^	2.13 ± 0.08 ^gA^
Kaempferol	3.10 ± 0.21 ^hA^	1.38 ± 0.04 ^hB^
Isorhamnetin	3.69 ± 0.21 ^gA^	0.60 ± 0.02 ^hB^
Total	273.44 ± 5.01	95.93 ± 1.58

Values expressed as µg/g of flour (dry matter). ND: non detected. For each flour, values with identical lowercase letter indicate significant differences (*p* < 0.05). For the same compound, values with identical capital letter indicate significant differences (*p* < 0.05).

**Table 5:** Antioxidant properties of almond skin flours obtained from *Turrón* coproducts.

Sample	DPPH^•^ (mg TE/g Flour)	ABTS^•+^ (mg TE/g Flour)	FRAP (mg TE/g Flour)	FIC (mg EDTA/g Flour)
ASFF ^1^	0.46 ± 0.41 ^b^	5.35 ± 0.60 ^b^	2.94 ± 0.70 ^a^	0.12 ± 0.01 ^b^
ASFC	2.28 ± 0.05 ^a^	15.81 ± 1.20 ^a^	3.34 ± 0.24 ^a^	0.21 ± 0.01 ^a^

^1^ For the same assay, different lowercase letters indicate significant differences (*p* < 0.05). DPPH^•^: 2,2-diphenyl-1-picrylhydrazyl radical scavenging assay; ABTS^•+^: 2,2′-azinobis-(3-ethylbenzothiazoline-6-sulfonic acid) radical scavenging assay; FRAP: Ferric reducing antioxidant power; FIC: Ferrous ion-chelating ability assay.

## Supplementary Materials

The following are available online at https://www.mdpi.com/2304-8158/9/6/727/s1, Table S1: Precursor ions (*m*/*z*), fragment ion transitions and main UHPLC-MS/MS optimized parameters (DP: declustering potential; EP: entrance potential; CE: collision energy; CXP: collision cell exit potential).
