# Supplementary material for: Turrón Coproducts as Source of Bioactive Compounds: Assessment of Chemical, Physico-Chemical, Techno-Functional and Antioxidant Properties
Source: Foods. 2020 Jun 3;9(6):727. doi: 10.3390/foods9060727 (PMC7353530; doi:10.3390/foods9060727)
Supplement: Supplementary file 1 [file foods-09-00727-s001.pdf]

Table S1. Precursor ions (*m/z*), fragment ion transitions and main UHPLC-MS/MS optimized parameters (DP: declustering potential; EP: entrance potential; CE: collision energy; CXP: collision cell exit potential).

**Compound**	***m/z***	**Fragment ion transition**	**DP**	**EP**	**CE**	**CXP**
Protocatechuic acid	151	107	-37	-13	-10	-3
4-Hydroxybenzoic acid	137	93	-9	-13	-18	-6
Catechin	289.2	123	-12	-6	-14	-4
Vanillic acid	167	151.3	-50	-6	-18	-5
Caffeic acid	179	135	-50	-5	-21	-5
Ferulic acid	193	134	-52	-9	-21	-4
O-coumaric acid	162.6	119	-29	-6	-19	-8
Sinapic acid	222.9	163.9	-61	-5	-21	-5
Syringic acid	197	120.8	-44	-10	-23	-8
Cinnamic acid	169	125	-51	-10	-20	-4
Chlorogenic acid	353.1	191	-14	-11	-23	-6
epicatechin	441	169	-15	-10	-22	-6
p-coumaric acid	162.6	119	-29	-6	-19	-8
Quercetin-3-rutinoside	609.4	301	-35	-10	-45	-5
Quercetin-3-glucoside	463	300	-120	-10	-30	-18
Kaempferol-3-rutinoside	593.1	285	-134	-5	-50	-12
Kaempferol-3-glucoside	447.1	284	-26	-8	-40	-12
Isorhamnetin-3-rutinoside	625.18	308.2	-29	-7	-50	-12
Quercetin	301	151	-39	-10	-24	-14
Kaempferol	285	288.7	-160	-10	-40	-7
Isorhamnetin	317	117	-91	-9	-48	-7
